# Lycopene/Arabinoxylan Gels: Rheological and Controlled Release Characteristics

**DOI:** 10.3390/molecules17032428

**Published:** 2012-02-28

**Authors:** Ana B. Hernández-Espinoza, Marina I. Piñón-Muñiz, Agustín Rascón-Chu, Víctor M. Santana-Rodríguez, Elizabeth Carvajal-Millan

**Affiliations:** 1 Chemistry Faculty, Autonomous University of Chihuahua, Chihuahua 31125, Mexico; Email: vsantana@uach.mx (A.B.H.-E.); vsantana@uach.mx (M.I.P.-M.); vsantana@uach.mx (V.M.S.-R.); 2 CTAOV, Research Center for Food and Development, CIAD, AC., Hermosillo, Sonora 83000, Mexico; 3 CTAOA, Laboratory of Biopolymers, Research Center for Food and Development, CIAD, AC., Hermosillo, Sonora 83000, Mexico; Email: ecarvajal@ciad.mx

**Keywords:** lycopene, ferulated arabinoxylans, gelling, controlled release

## Abstract

Arabinoxylan gels exhibiting different rheological and lycopene transport properties were obtained by modifying the polysaccharide concentration from 3 to 4% (w/v). The apparent lycopene diffusion coefficient decreased from 2.7 × 10^−7^ to 2.4 × 10^−7^ cm^2^/s as the arabinoxylan concentration in the gel changed from 3 to 4% (w/v). A low amount of lycopene is released by diffusion from arabinoxylan gels. These results indicate that arabinoxylan gels could be carriers for lycopene delivery in specific sites after network degradation. The possibility to modulate lycopene release from arabinoxylan gels makes these biomaterials potential candidates for the controlled delivery of biomolecules.

## 1. Introduction

Gels are polymeric three-dimensional networks, which swell on contact with water but do not dissolve [1]. The water absorption property enables gels for applications like food additives, enzyme immobilization and controlled release of active compounds. Gels have been used as controlled release matrices in the food, medicine, agronomy and cosmetic industries [2]. Although most studies concern gels made from synthetic polymers, gellable native or tailored polysaccharides, generally non-toxic and highly biocompatible, are receiving increasing attention [3]. Polysaccharide gels can be used as matrices to control the release of functional components. Some polysaccharide networks can protect entrapped molecules while passing through stomach and small intestine, releasing them in the colon during gel degradation [1].

Studies on the utilization of polysaccharide gels as controlled release matrices usually involve chitosan, alginate, modified dextran and starch derivatives [2]. Other polysaccharides such as arabinoxylans have been studied to a minor extent. Arabinoxylans are non-starch polysaccharides from the cell walls of cereal endosperm constituted by a linear backbone of β-(1→4)-linked xylose units containing α-L-arabinofuranosyl substituents attached through O-2 and/or O-3 [4]. Arabinoxylans can present some arabinose residues ester-linked on (O)-5 to ferulic acid (FA, 3-methoxy-4-hydroxy-cinnamic acid), being called “ferulated”. Ferulated AX can gel by covalent cross-linking involving FA oxidation by some chemical or enzymatic (laccase and peroxidase/H_2_O_2_ system) free radical-generating agents [5]. Diferulic acids (di-FA) [6] and tri-ferulic acid (tri-FA) [7] have been identified as covalent crosslinked structures in laccase gelled arabinoxylans. Both covalent bridges (diFA, tri-FA) and physical interactions between AX chains have been reported to be involved in the arabinoxylan gelation process and the final gel properties [7]. AX gels present interesting properties like neutral taste and odour, high water absorption capacity (up to 100 g of water per gram of dry polymer) and absence of pH, electrolyte and temperature susceptibility [4], which confer them potential applications as a delivery matrix.

Lycopene is an important biological compound found mainly in tomatoes. This molecule has received increasing attention because of its possible role in the prevention of chronic diseases such as atherosclerosis, skin cancer, prostate cancer and colon cancer [8]. However, lycopene can be susceptible to oxidation, especially when stored in the presence of oxygen [9]. Therefore, entrapment of lycopene in polymeric networks could be an alternative to reduce lycopene oxidation. To our knowledge, lycopene entrapment and release capability of arabinoxylans gels has not been reported elsewhere. The objective of this research was to investigate the entrapment and release of lycopene from arabinoxylan gels exhibiting different rheological characteristics.

## 2. Results and Discussion

### 2.1. Lycopene Analysis

The lycopene content in the sample used in the present study was 438 µg/g of tomato dry basis, which is in the range reported for several tomatoes varieties and tomato products (from 6.6 to 490 µg/g) [10]. It is known that lycopene is the predominant pigmented compound in red tomatoes, but other carotenoids such as carotene and lutein constitute about 20% of the total carotenoids in fresh red tomato tissue [11]. In this regard, it has been suggested that on the basis of the levels and the 560 nm extinction coefficients of these minor carotenoids, the contribution of these compounds will result in an overestimation of lycopene content by less than four percent [12].

### 2.2. Lycopene Entrapment

**Figure 1:**
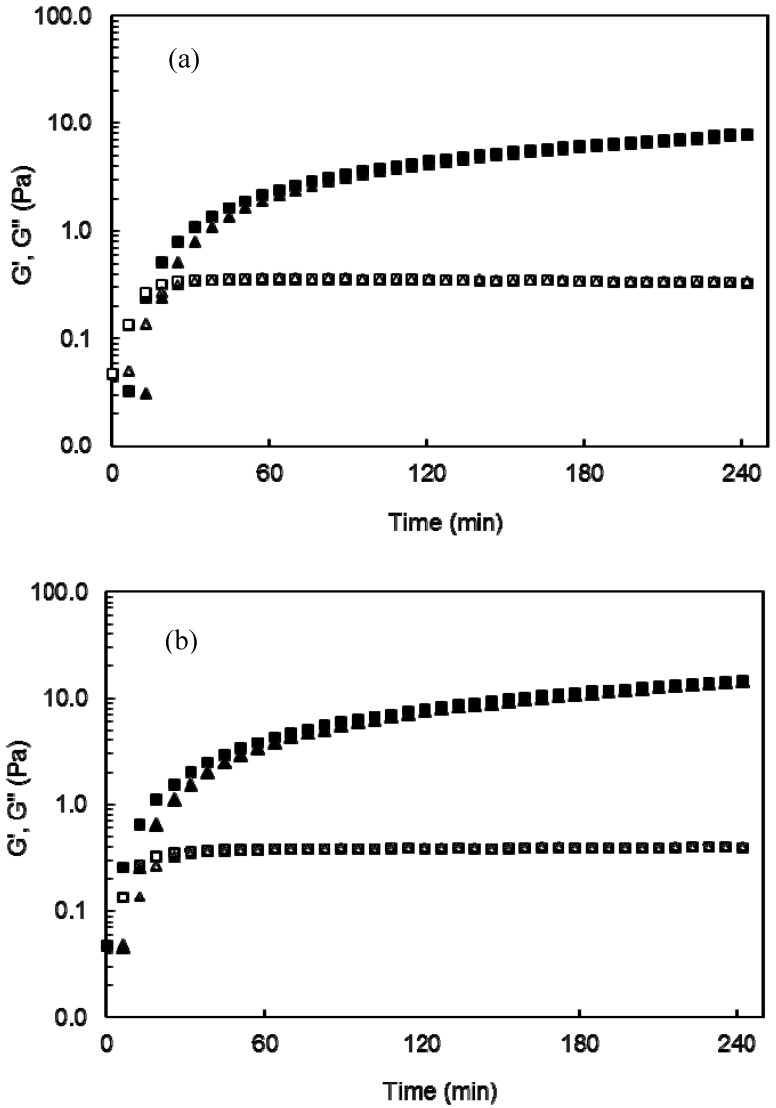
Monitoring the storage (G’) and loss (G”) modulus of arabinoxylan (G’ ■, G” ◻) and arabinoxylan-lycopene (G’ ▲, G” ∆) solutions at (**a**) 3% and (**b**) 4% (w/v) in polysaccharide during gelation by laccase at 25 °C, 0.25 Hz and 5% strain.

Laccase-treated arabinoxylan/lycopene mixtures were monitored by small deformation dynamic rheology. For all arabinoxylan/lycopene treatments the storage (G’) and loss (G”) modulus rose to reach a pseudo plateau (Figure 1).

**Figure 2:**
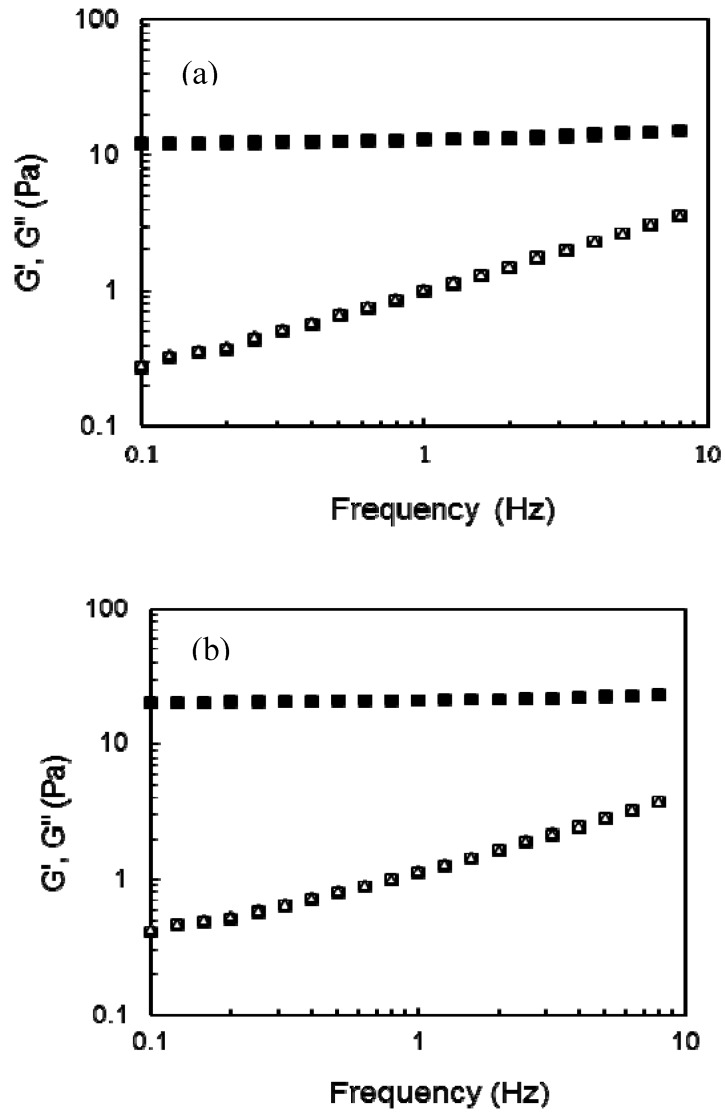
Mechanical spectrum of arabinoxylan (G’ ■, G” ◻) and arabinoxylan-lycopene (G’ ▲, G” ∆) gels at (**a**) 3% and (**b**) 4% (w/v). Data obtained at 25 °C and 5% strain.

At the end of gelation (4 h) G’ and G” values were 9 and 0.3 Pa and 20 and 0.4 Pa for gels at 3 and 4% (w/v) in arabinoxylans, respectively. No gelation was detected in arabinoxylan and arabinoxylan/lycopene mixtures without laccase addition. In the present study 12.5 and 16.0 mg of lycopene per g of arabinoxylans have been entrapped for gels at 3 and 4% in arabinoxylans, respectively. A previous study reported 0.1 mg of lycopene entrapped per g of gelatin/polyglutamic acid as carrier [13], but in that study the rheological characteristics of the gels were not investigated. On the other hand, most of non modified polysaccharides form physical gels sensible to temperature, ionic strength or pH changes while arabinoxylans gels are covalent networks which are not affected by these factors [4]. In addition, it is possible that higher lycopene amounts could be entrapped in arabinoxylan gel as in the present study G’ values of the networks were not affected and differences in the gel mechanical properties are determinant in their practical use. The mechanical spectra of arabinoxylan and arabinoxylan-lycopene gels after 4 h gelation (Figure 2) was typical of solid-like materials with a linear G’ independent of frequency and G” much smaller than G’ and was dependent on frequency [14]. This behavior is similar to that previously reported for arabinoxylan gels [4].

### 2.3. Controlled Release of Lycopene

**Figure 3:**
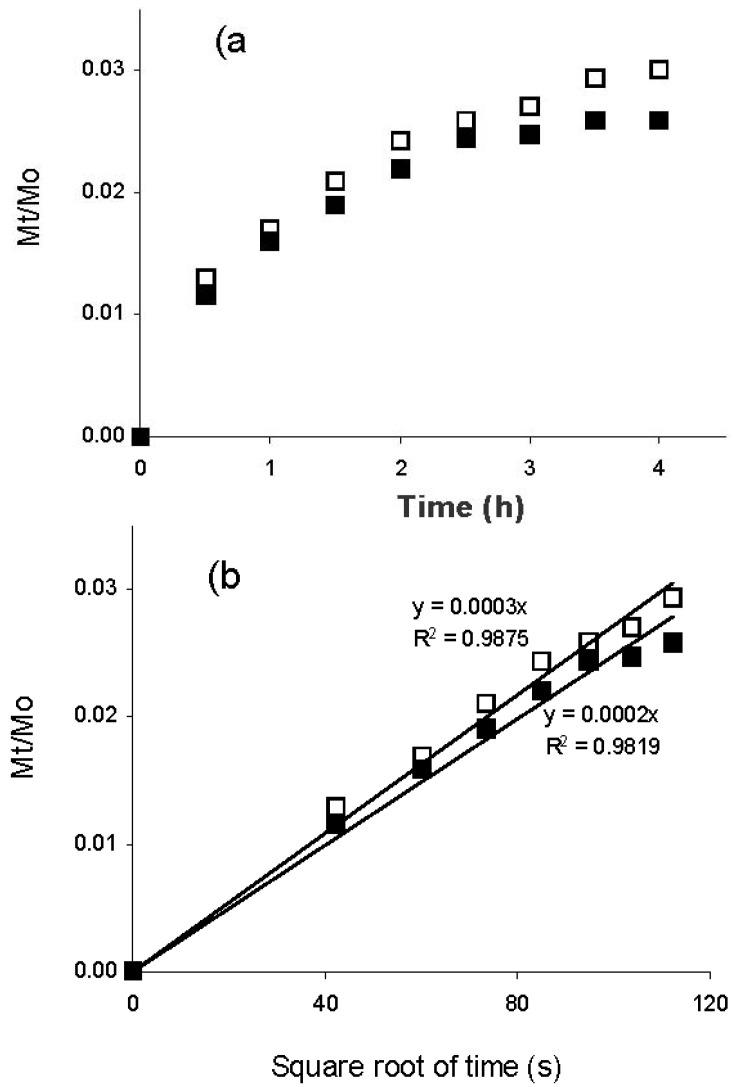
Cumulative release (Mt/Mo) of lycopene from arabinoxylans gels at (◻) 3% and (■) 4% (w/v) in polysaccharide: (**a**) as a function of time; and (**b**) as a function of root time. Lycopene release was followed at 25 °C and 90 rpm during 4 h.

The kinetics of lycopene release from arabinoxylan gels are shown in Figure 3a. Linear relationships between cumulative release (Mt/Mo) and the square root of time were found for lycopene release from arabinoxylan gels, allowing the calculation of the apparent diffusion coefficients (Dm) of this compound from the gels (Figure 3b). The rate of lycopene release from arabinoxylan gels was dependent on the polysaccharide concentration. The apparent diffusion coefficient was 2.7 × 10^−7^ and 2.4 × 10^−7^ cm^2^/s for lycopene in arabinoxylan gels at 3 and 4% in polysaccharide, respectively (Table 1). A previous study [15] reported a higher apparent diffusion coefficient value for lycopene in a tomato juice/soy mixture, which could be attributed to a faster lycopene mobility in a liquid phase in comparison to those registered in arabinoxylan gels.

**Table 1:** Release parameters of lycopene from arabinoxylan gels.

Arabinoxylan concentration (% w/v)	Dm × 10^−7^ (cm^2^/s)	Lycopene released (%) ^a^
3.0	2.7 ± 0.10	3.7 ± 0.2
4.0	2.4 ± 0.10	2.6 ± 0.3

All values are average from three replicates: ^a^ Weight of lycopene released after 4 h of gel incubation/weight of lycopene entrapped in the gel.

As presented in Table 1, the percentages of lycopene released by the end of the test (4 h) were 3.7 and 2.6% for gels at 3 and 4% in arabinoxylans, respectively. These results become of importance when it is desirable to protect the carried lycopene from the gastric environment for further release inside intestinal lumen and promote their uptake after gel degradation by colonic bacteria. It has been previously reported [16] that maize arabinoxylans gels are degraded to oligosaccharides by bacteria from the colon. In the present study, gel incubation was fixed at 4 h as the average transit time from oral ingesta to the colonic region. The total amount of lycopene released during 4 h was minimal and thus possibly more than 96% of this compound could reach the intestinal region where it is expected to act.

## 3. Experimental

### 3.1. Materials

Maize bran arabinoxylans were obtained and characterized as previously described [17]. They presented an A/X ratio of 0.8 and a ferulic acid content of 0.34 µg/mg. The relative percentages of each di-FA structure were: 16, 21 and 63% for the 8-5’, 8-O-4’ and 5-5’ structures, respectively. All chemical products were purchased from Sigma Chemical Co. (St. Louis, MO, USA).

### 3.2. Methods

#### 3.2.1. Lycopene Extraction and Quantification

Tomatoes (*Lycopersicon esculentum* Mill.) cvar. Sedona were kindly provided by a commercial greenhouse in Northern Mexico. Fresh tomato samples were homogenized and then diluted in deionized water to produce an uniform slurry. The extraction and quantification of lycopene was performed as reported before [10]. A UV-Visible spectrophotometer at 503 nm was used to estimate the lycopene content.

#### 3.2.2. Preparation of Arabinoxylans Gels and Arabinoxylans-Lycopene Gels

Arabinoxylan solutions at 3 and 4% (w/v) and arabinoxylan-lycopene mixtures at 3 or 4% (w/v) in polysaccharide containing 12.5 and 16.0 mg of lycopene per g of arabinoxylans were prepared in 0.05 M citrate phosphate buffer at pH 5 containing sodium taurocholate and Triton X-100. Laccase (1.675 nkat/mg arabinoxylan) was used as cross-linking agent. Gels were allowed to form for 4 h at 25 °C.

#### 3.2.3. Rheological Measurements

The formation of the arabinoxylan gel was followed using a strain-controlled rheometer (AR-1500ex, TA Instruments, AR1500ex, TA Instruments, New Castle, DE, USA) in oscillatory mode as reported before [7]. Arabinoxylan gelation was studied for 4 h at 25 °C. Arabinoxylan solutions were mixed with laccase and immediately placed in the cone and plate geometry (5.0 cm in diameter, 0.04 rad in cone angle) maintained at 25 °C. Exposed edges of the sample were covered with mineral oil fluid to prevent evaporation during measurements. Arabinoxylan gelation kinetics was started monitored at 25 °C for 4 h by following the storage (G’) and loss (G”) modulus. All measurements were carried out at 0.25 Hz and 5% strain. From strain sweep tests, arabinoxylan gels showed a linear behavior from 1.5 to 10% strain. The mechanical spectra of gels were obtained by frequency sweep from 0.1 to 10 Hz at 5% strain and 25 °C.

#### 3.2.4. Lycopene Release

Arabinoxylan-lycopene mixtures (2 mL) were poured into a 30 mL cylindrical plastic cell (diameter 30 mm) just after laccase addition. Arabinoxylan-lycopene gels were allowed to form during 4 h at 25°C. Then, lycopene was released in 0.02% (w/v) sodium azide solution (6 mL) containing sodium taurocholate and triton X-100 placed on the gel surface. Gels were incubated at 25 °C and 90 rpm tangential rotation and liquid medium was renewed every 30 minutes from 0.5 to 4 h. At the end of the test, the gels were hydrolyzed as described before [18] in order to quantify un-released lycopene. Lycopene recovery (released lycopene + un-released lycopene) was near to 100%. The lycopene was quantified as described elsewhere [10]. Lycopene release was characterized by calculating an apparent diffusion coefficient (Dm). This Dm was estimated from the release kinetics curve, fitted by using an analytical solution of the second Fick’s law [19], which gives the solute concentration variation as a function of time and distance:

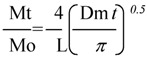

where Mt is the accumulated mass of lycopene released at time t, Mo is the mass of lycopene in the gel at time zero, L is the sample thickness (0.4 cm) and Dm is the diffusion coefficient. By plotting the relative solute mass released (Mt/Mo) at time t *versus* the square root of time, a simplified determination of Dm can be made assuming that Dm is constant and that the sample is a plate with a thickness L. In this study, the apparent Dm was calculated from the linear part of the Mt/Mo *vs.* time curves [18]. The percentage of lycopene released at the end of the test was also calculated. 

### 3.3. Statistical Analysis

All measurements were made in triplicate and the coefficients of variation were lower than 10%. Results are expressed as mean values.

## 4. Conclusions

The cross-linking method used allowed the formation of arabinoxylan gel in the presence of lycopene without modifying the rheological properties of the gel. The lycopene release rate and quantity are dependent on the polysaccharide concentration in the gel. A low amount of entrapped lycopene is released by diffusion while most of this compound would be liberated only after gel degradation. These results indicate that arabinoxylan gels could be carriers for lycopene delivery in specific sites after network degradation. Additional studies will be required in order to understand the effect of different lycopene concentrations in the diffusion coefficient value.
